# Methodological Encounters with the Phenomenal Kind

**DOI:** 10.1111/j.1933-1592.2010.00483.x

**Published:** 2012-03

**Authors:** Nicholas Shea

**Affiliations:** Somerville College, University of Oxford

## Abstract

Block’s well-known distinction between phenomenal consciousness and access consciousness has generated a large philosophical literature about putative conceptual connections between the two. The scientific literature about whether they come apart in any actual cases is rather smaller. Empirical evidence gathered to date has not settled the issue. Some put this down to a fundamental methodological obstacle to the empirical study of the relation between phenomenal consciousness and access consciousness. Block (2007) has drawn attention to the methodological puzzle and attempted to answer it. While the evidence Block points to is relevant and important, this paper puts forward a more systematic framework for addressing the puzzle. To give it a label, the approach is to study phenomenal consciousness as a natural kind. The approach allows consciousness studies to move beyond initial means of identifying instances of the kind like verbal report, and to find its underlying nature. It is well-recognised that facts about an underlying kind may allow identification of instances of the kind that do not match the initial means of identification (cp. non-liquid samples of water). This paper shows that the same method can be deployed to investigate phenomenal consciousness independently of access consciousness.

## 1. Introduction: a Distinctive Methodological Problem

Block’s important distinction between phenomenal consciousness (P) and access consciousness (A) (Block 1995, 2001) has generated a large literature about the ‘hard’ problem, necessary connections and explanatory gaps. More recently it has been suggested that there is a separate, methodological problem: even if physicalism is unproblematically true, scientific methods will be unable to differentiate P-consciousness from A-consciousness empirically. It is argued that even if there really are cases of P without A, or the converse, science will never be able to discover them.

The problem can be sketched as follows. The scientific study of P-consciousness has to start with some cases of P to form an object of study, together with some instances of ¬P by which to identify what is distinctive about the P cases. The thought is that these initial instances will all be cases where conscious contents are also access conscious, so all the cases of P will also be cases of A, and ¬P of ¬A. The claim is that this presents a methodological challenge: that if there are cases of P without A (or the converse), we could never discover them.

The methodological challenge has been given prominence by Block’s recent attempt to answer it (Block 2007). It is not just a philosophers’ curiosity. It’s force is felt by many working scientists, leading some to express humility about their power to investigate phenomenal consciousness. Typically, they take themselves instead to be capable only of studying the mechanisms of global accessibility of information:

‘[we] aim at characterising the crucial differences between those aspects of neural activity that can be reported by a subject, and those that cannot. … conscious access is one of the few empirically tractable problems presently accessible to an authentic scientific investigation.’ (Dehaene & Changeux 2004, p. 1146)

In the light of the impact of this thought on actual scientific practice, and of the sceptical responses to Block’s treatment,^1^ there is a pressing need for a methodological approach that is capable of separating P from A empirically. That is the goal of this paper. To give it a label, the approach is to study phenomenal consciousness as a natural kind (the ‘phenomenal kind’). The result will not be a positive case for the existence of P without A or A without P, or for their always being co-instantiated, but the prescription of a systematic way of gathering evidence to answer the question.

Some versions of the problem define A-consciousness in a way that rules out studying it as a natural kind. Section 2 argues that the enquiry should not be foreclosed in this way and goes on to pinpoint a conception of the A property that does generate a genuine empirical question for which a methodological issue can arise. Section 3 identifies a beguiling thought, the initial plausibility of which underpins much of the intuitive force of the methodological challenge. That thought is a bad one if phenomenal properties could be natural kind properties. The real methodological challenge is identified in Section 4. It arises because we are antecedently uncertain whether cognitive access is constitutively connected to phenomenality. Section 5 sets out a framework equal to that challenge and explains how the natural kind methodology will generate evidence that is likely to resolve it. If the framework produces evidence that there is only one underlying natural kind, we can conclude that P and A are always co-instantiated. If two underlying natural kinds are uncovered, that is some evidence that P and A come apart, but the picture is more complicated. Section 6 identifies some further steps that could then be taken to bolster the evidence for P without A.

## 2. A formulation of A-Consciousness that Generates a Genuine Empirical Question

### 2.1 P_p_

To assess the prospects for distinguishing P from A empirically, we need to be clearer about which properties we are picking out. In the course of section 2 we set aside some ways of formulating the A property that fail to generate an empirical question about which there could be a methodological challenge and thereby arrive at a way of understanding A that makes distinguishing between P and A a genuinely empirical problem.

We start in this subsection with the property of being phenomenally conscious. If there is such a property, it is shared by all the mental episodes which have a ‘what it’s likeness’ to them. Each such episode also has its own phenomenal character, a determinate way of being phenomenally conscious. The methodological issue arises in the same way for both the determinates and for the putative determinable property P. Since it is possible to doubt the existence of the determinable while accepting the determinates (Shea & Bayne 2010, p. 476) we deal with the latter, for example the phenomenal property a person instantiates when she is phenomenally conscious of a red cube against a white background. We adopt the usual practice of using contents or schematic letters for contents to pick out phenomenal properties, without presupposing representationalism or conceptualism about the phenomenal character of experience. We use P_p_ for the property of being phenomenally conscious that p. To focus on the methodological issues, we are assuming physicalism: P_p_ is some physical property (intrinsic, extrinsic or functional) of the subject of the experience.

The science of consciousness must start with some instances of the phenomenon. Four sources of evidence are relatively uncontroversial. They are defeasible in certain cases. As the enquiry proceeds we will learn more about why each is a source of evidence about P_p_, which may lead us to revise the probative value we attach to them, perhaps radically. But they are adequate to identify instances of the phenomenon for further investigation.

The first is obvious: S’s verbal report that p (based on her seeing, hearing, feeling, etc., that p).^2^ We also take S’s negative verbal reports (‘there was no red cube’) as evidence that S was not phenomenally conscious that p. Second, many experiments rely on S’s use of the information that p to make a voluntary perceptual discrimination, which is then reported by a non-verbal action like a button press. These are ‘reports’ in some extended sense, since subjects are given and agree to verbal instructions about what to do, and are also typically asked afterwards (often informally) to check that they were making their discriminations in a standard, conscious manner (unlike priming, say). Third, we infer that S is phenomenally conscious that p when S uses the information that p to plan and carry out other voluntary actions. Finally, we infer that S was phenomenally conscious that p when presented with a stimulus if, on a subsequent occasion, she can consciously remember the stimulus (which in turn is evidenced by one of the foregoing tests).

These categories are not exclusive, e.g. verbal report is a form of voluntary action. The second and third types of evidence give us some initial traction on consciousness in cases where verbal report is unavailable (e.g. aphasia) or impossible (e.g. infants and other animals). Some theorists may want to enlarge upon these initial lines of evidence. Others would restrict them. Neither move undermines the structure of what follows.

### 2.2 A Priori Connections

In this subsection we will reject two formulations of the A property which presuppose that phenomenal consciousness cannot be studied as a natural property.

The initial lines of evidence all involve the subject’s having some kind of personal-level access to the content p. Some claim that there is an a priori necessary connection between phenomenality and these forms of cognitive access. For example, Chalmers argues that the study of consciousness depends on there being ‘epistemic levers’, which are established a priori, by which instances of phenomenal consciousness can be identified (Chalmers 1996). One is that A → P, as a matter of epistemic necessity (pp. 218–22, 233–46).

If that were all we knew about P, then we would indeed have no way of knowing whether non-A cases were P or not. However, Chalmers thinks that we can also rely on an a priori bridge principle in the other direction: P → A. These bridge principles only express epistemic necessities, so they are consistent with the metaphysical possibility of P without A (Chalmers 1996, pp. 221–2, 242–6, Chalmers 1998, pp. 6). However, if we do indeed know a priori that these ‘epistemic levers’ express epistemic necessities, then it is built-in that no knowledge of cases of P without A is possible.

Chalmers has his own reasons for embracing these a priori bridge principles. However, in order to focus on the claim that there is a specifically methodological problem, we should not presuppose at the outset that there is some A property such that it is impossible to know about cases of P that are not also cases of A. If P_p_ is a natural property then its character may differ from or go beyond the ways in which we are inclined to judge its presence. It is a familiar point that natural properties are normally verification-transcendent. Even if our initial means of identifying water rely on its being a transparent odourless potable liquid, we can go on to identify instances of that natural property which do not match our initial means of identification (e.g. frozen H_2_O).

So that we don’t rule out the start of the enquiry the possibility that P_p_ is a natural property, we should take the initial lines of evidence listed above simply to be defeasible ways of identifying instances of P_p_. They can act as ‘epistemic levers’ that get the inquiry going without assuming any necessary connections (epistemic, nomological or metaphysical) between A and P. If we are to investigate P_p_ in a way that remains open to both outcomes — that there are necessary connections between access and phenomenality, and that there are not — we cannot build in a priori necessary connections at the outset, whether metaphysical or epistemic.

Chalmers’ position is rather more subtle, because he allows that our understanding of cognitive accessibility may be revised. The bi-directional bridge principles connecting accessibility and phenomenality only remain in place because A is revised so as to be the best functional correlate of P. If that is how accessibility is thought of, then it is again guaranteed that if there are cases of phenomenality without accessibility, we cannot know about them. There can be no cases where we are epistemically justified in thinking there is P without A or A without P because, by construction, in such cases we would revise our conception of A to make the two terms co-extensional. That way of proceeding would still leave open the interesting issue of whether phenomenality is identical to the functional property A, or to the categorical basis of A (Papineau 2007, ch. 7). But it still suffers from the problem of ruling out at the outset the possibility that P_p_ is a verification-transcendent natural property.

In short, if there are these kinds of a priori necessary connections between phenomenality and accessibility (either with our initial lines of evidence or some polished-up conception of accessibility), then there is no genuine empirical question about whether they come apart in actual cases, about which a distinctively methodological problem could arise. The methodological challenge only comes into view when we leave the possibility open, at the outset of the enquiry, that initial lines of evidence (verbal report, etc.) are defeasible ways of identifying a verification-transcendent natural property P_p_.

### 2.3 A_p_

Distinguishing between a natural property and ways of identifying it does not put an end to the matter. Cognitive access generates a special problem. A is not just a motley collection of every kind of correlational evidence about phenomenality. It is supposed to be some kind of information-processing property that is capable, itself, of accounting for those lines of evidence (the capacity to give a verbal report that p, etc.).

The fact that each of the four lines of evidence we started with is taken independently to be evidence of the presence of the very same property, P_p_, suggests that they should converge. As indeed we find. If a subject is identified as phenomenally conscious that p according to one of the lines of evidence, she tends to satisfy the others. If S verbally reports that p on the basis of perception, she would also use the information that p to plan and carry out voluntary actions, and so on. We have already noted that there are cases in which these lines of evidence diverge; for example in aphasics the evidence from verbal report will not converge with the other three lines of evidence. Nevertheless, the lines of evidence do agree with one another across a wide range of ordinary cases.

But here’s the worry. Convergence raises the possibility of there being an information-processing mechanism which is responsible for that convergence. Notice that convergence occurs when the information that p is available for directing a wide range of S’s behaviours. It excludes cases where S’s sensitivity to the information that p is encapsulated within a particular mode of behaviour. So convergence immediately raises the prospect of there being some mechanism that makes the information that p available for directing a wide range of S’s behaviours — a mechanism of global availability of information. Some refer to such a mechanism as a global workspace (Dehaene & Naccache 2001). Information in the global workspace can be used by a range of consumer systems: language production, action planning, decision-making, episodic memory, etc.

Having a mechanism to make information globally available for directing a wide range of her behaviour (having a global workspace) is a putative property of the subject S. The mechanism will operate on different contents on different occasions. When the mechanism makes the information that p globally available for use in directing a range of behaviours, S has property A_p_:

S instantiates property A_p_ = _df_

(i) S has a mechanism M for making information directly^3^ available for use in directing a wide range of behaviours; and
(ii) M is making the information that p directly available for directing a wide range of potential behaviours of S.

S’s instantiating A_p_ would explain why she makes a verbal report that p, why she makes a voluntary perceptual discrimination based on p, why she uses the information that p to plan and carry out voluntary actions and why she can store the information that p in memory. Furthermore, instantiating A_p_ would also explain convergence — when she evinces one of these signs, she tends to display the others too. If access conscious contents are defined as those that are poised for ‘free use in reasoning and for direct “rational” control of action and speech’ (Block 1997, p. 382), a subject who instantiates A_p_ is thereby access conscious that p.

A_p_ is a contrary of informational encapsulation. In some cases, sensitivity to the information that p is exhibited in one, but only one, type of behaviour performed by a subject (e.g. the fine-grained information about object location encoded in the dorsal stream and used for online action guidance). Informational encapsulation explains why information is used by only one consumer system. In contrast, when S instantiates A_p_ the information that p is not encapsulated.^4^ Sensitivity to the information can be evinced in any type of behaviour, provided the consuming mechanisms giving rise to that behaviour are not otherwise disabled.

How does A_p_ generate a methodological challenge? Its potentially close evidential connection to P_p_ is a start. The four lines of evidence that we were taking to be initial evidence of the presence or absence of P_p_ are also very directly evidence of the presence or absence of A_p_, if there is such a property (and the converge of those lines of evidence is some evidence that there is). So the discovery of a mechanism A_p_ would immediately raise the question of its relation to P_p_: identity, necessary co-instantiation, the existence of P_p_ without A_p_, and so on.

It is worth emphasising that our A_p_ is rather different from the conceptions of access consciousness in the previous subsection which create an a priori bar to knowledge of phenomenality without access. Our A_p_ is a theoretical posit. It is not identical to the commonsense idea that some information is ‘accessible’ to the whole person, at the personal level. A_p_ is a property whose presence we will infer from the initial lines of evidence, supplemented by subsequent discoveries. The simplest way in which it could turn out that there is phenomenality without access in our sense is if we discover that there is no information processing property A_p_. Nevertheless, it is plausible that there is some common information processing mechanism that is responsible for the initial lines of evidence and their convergence. If so we want to know if it in fact dissociates from P_p_ and, if it does, how to differentiate empirically between instances of it and instances of P_p_.

### 2.4 The Empirical Question

We’ve seen that conceptions of phenomenal properties that tie them a priori to a set of criteria by which they are identified makes it impossible to distinguish them empirically from access properties. But a genuine empirical question does arise about the relation between P_p_ and a putative information-processing property A_p_ that is responsible for the initial lines of evidence of P_p_. On the one hand, is A_p_ just a characterisation of the underlying phenomenal property we were interested in, P_p_ or, if they’re not identical, is there some tight connection (at least nomological) such that they always go together in actual cases? On the other hand, are they separately instantiated in actual cases and, if so, how should we go about identifying such cases (cases of P_p_ without A_p_ or the converse)? Whether option one or option two obtains looks like it should be a straightforwardly empirical question.

There are several possibilities within each option. Option one encompasses the possibility that P_p_ is the realiser of A_p_, that they are co-instantiated as a matter of metaphysical necessity, and that they are identical. Option one also encompasses the possibility that nomological facts about how things are constituted — about how properties of parts and the relations of those parts fix properties of wholes as a matter of natural law — ensure that A_p_ and P_p_ are nomologically necessarily co-instantiated. If empirical results support option one, then deciding amongst the possibilities within option one is not a straightforwardly empirical matter.

Option two encompasses cases in which P_p_ is instantiated but not A_p_ (the usual focus) and cases in which A_p_ is instantiated but not P_p_. Even if it were a natural law that P_p_ unfailingly caused the instantiation of A_p_, they would come apart because P_p_ would be instantiated before A_p_ (barring some strange simultaneous causation).

Deciding between possibilities within an option is not straightforwardly empirical. However, deciding between the options should be. The methodological challenge is to say how the methods of science are adequate to that task. So, finally, we have arrived at a formulation of an A-property that gives rise to a genuine empirical question. The question is predicated on it turning out that subjects do instantiate the property A_p_ identified above (as to which there is reasonable evidence, sufficient to make the question of interest).

Empirical Options

(i) P_p_ and A_p_ are always co-instantiated.
(ii) There are subjects S and times t such that:
  (a) P_p_(S)-at-t and ¬{A_p_(S)-at-t}; or
  (b) A_p_(S)-at-t and ¬{P_p_(S)-at-t}.

**Figure 1:**
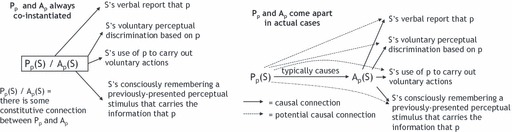
Relations between P_p_ and A_p_.

The methodological challenge is to show that deciding between (i) and (ii) is susceptible to empirical investigation at all. Although there are several ways that each option could be true, the leading candidates are as follows: [Fig. 1]

Both kinds of arrows represent direct causal links. On the supposition that we discover an information-processing mechanism for global availability, it is plausible that many or most of the behaviours used as initial tests of phenomenality would be caused by A_p_. On the left: P_p_ is always co-instantiated with A_p_, and so is as tightly connected to the behavioural signs as is A_p_ (P_p_ will actually be a cause, if P_p_ = A_p_). On the right: P_p_ typically causes the information that p to be made globally available, and thereby causes the behavioural signs of consciousness. However, we do not exclude the possibility that being phenomenally conscious that p might directly cause particular behavioural outcomes, unmediated by the information becoming *globally* available. Those potential causal connections are represented by broken arrows on the right.

## 3. A Beguiling Thought Dismissed

This section identifies a beguiling thought which underpins much of the intuitive force of the methodological challenge. It may derive from thinking about access consciousness in one of the ways above that rules out at the outset the possibility of knowing about phenomenality without access. In this section we will see that the force of the beguiling thought evaporates once we formulate A_p_ in a way that produces a genuine empirical question and leaves open the possibility that P_p_ is a separate natural property.

The beguiling thought is that our way of identifying instances of P_p_ via A_p_ must be the most secure, on pain on undermining the enquiry. Surely, the thought runs, if taking A_p_ to be good evidence of P_p_ is the very basis on which we come to identify further evidential tests for the P_p_ phenomenon, we could never think that those further tests were more reliable tests of P_p_ than is A_p_ itself, or the whole basis of the inquiry would be undermined? As Block puts it, ‘any evidence would inevitably derive from the reportability of a phenomenally conscious state, and so it could not tell us about the phenomenal consciousness of a state which cannot be reported’ (2007, p. 483). How could an investigation wholly founded on the idea that the presence or absence of A_p_ is good evidence for the presence and absence of P_p_ give rise to a test according to which a subject lacking property A_p_ nevertheless instantiated P_p_?

That way of putting the challenge presupposes that our initial lines of evidence are only evidence of P_p_ because they are evidence of A_p_ which is in turn evidence for P_p_. But we saw above that, if we are not ruling it out that phenomenality and access could be separate natural properties, we should take the initial lines of evidence as evidence for P_p_ irrespective of their connection with A_p_; they would be evidence for P_p_ even if we discover there is no such property as A_p_. So we should reject the idea that the evidential force of the initial lines of evidence derives from the evidential force of A_p_ as evidence for P_p_.

Nevertheless, if there is a mechanism of global availability of information, then it is likely that the initial lines of evidence like giving a verbal report are caused by the subject’s instantiating A_p_. (We are not ruling it out that a subject could display evidence of P_p_, for example by using the information that p to plan a voluntary action, without instantiating A_p_ even though she has a mechanism of conscious access — a possibility we return to below.) If all or most of the initial behavioural tests are caused by A_p_, then instantiating A_p_ must also be strong evidence that the subject instantiates P_p_.

If we start with our initial lines of evidence and discover a mechanism A_p_ that mediates them, could we ever come up with stronger evidence for P_p_ than the instantiation of A_p_, or stronger evidence for ¬P_p_ than ¬A_p_? Working with instances of P_p_ identified through our initial means of identification, we generate another test T_p_ for the presence of P_p_. Let’s say that test T_p_*trumps* A_p_ iff T_p_ disagrees with A_p_ about the presence or absence of P_p_*and* we rely on property T_p_ over property A_p_ as diagnostic of the presence or absence of P_p_. Trumping is a matter of what we take the relative strength of the two sources of evidence to be. T_p_ can trump A_p_ in one of two ways:

T_p_ trumps A_p_ iff

(a) T_p_(S) & ¬A_p_(S) is good evidence that P_p_(S); or
(b) ¬T_p_(S) & A_p_(S) is good evidence that ¬P_p_(S).

The beguiling thought is that, in an enquiry founded on identifying instances of P_p_ by means which all depend causally on A_p_, if empirical study of those instances were to generate a test T_p_ that trumps A_p_, that would be inconsistent with our starting assumption, thereby undermining the whole inquiry.

Chalmers appears in places to be motivated by something like this thought. He starts with the claim that, to get evidence of a link between a neural process N and consciousness we must already have evidence of a link between N and his A-consciousness property, ‘awareness’ (Chalmers 1996, p. 239). He goes on to argue as follows:

‘It is very plausible that some kind of awareness is necessary for consciousness. Certainly, all the instances of consciousness that I know about are accompanied by awareness. There seems to be little reason to believe in any instances of consciousness *without* the accompanying functional processes. If there are any, we have no evidence for them, not even indirect evidence, and we could not in principle. It therefore is reasonable to suppose on the grounds of parsimony that wherever there is consciousness, there is awareness. If we are wrong about this — if for example a static electron has the rich conscious life of a Proust — then we will certainly never know about it.’ (Chalmers 1996, p. 243–4)

Chalmers’ position makes sense if access is defined in one of the ways that we set aside in the last section. But does the argument still run if we leave open the possibility that P_p_ and A_p_ are natural properties, and that the question whether they dissociate is an empirical one? The idea that the initial basis of our investigation of a phenomenon must remain secure has some superficial plausibility, but does not survive close scrutiny. To see why not, we should start with a precise statement of the supposed challenge:

The beguiling thought

At the start of enquiry, all cases that are studied empirically as instances of P_p_ are identified as such because they have property A_p_ (and all instances of ¬P_p_ because ¬A_p_): initially there is no evidence for instances of P_p_ that do not have property A_p_ or instances of A_p_ that do not have property P_p_. Therefore, we could not, even in principle, have any direct or indirect evidence that a subject S is such that P_p_(S) & ¬A_p_(S) or such that ¬P_p_(S) & A_p_(S).

The idea that this is a general principle that applies whatever properties are being studied is superficially plausible, but false. This history of science if full of counterexamples (e.g. water / H_2_O). Our tactic in the rest of the paper will be first, to spell out carefully how science does manage to transcend initial lines of evidence in other cases, and second, to show that there is no bar to deploying the same method in the study of phenomenal consciousness. In my view quite a lot of the discussion of the methodological difficulty of studying P_p_ in the presence of A_p_ can be traced to the idea that the beguiling thought is a general truth.

An example we will return to below illustrates how things go with ordinary sciences. Genes were initially identified by means of the now-familiar signature of Mendelian inheritance, with traits passing down the generations in patterns that seem to depend on the particulate inheritance of dominant or recessive genes for those traits. Genes were hypothesised as being the causal basis of these patterns of inheritance. It then took years of investigation to discover that DNA was the basis of these patterns of inheritance. But DNA-based inheritance is far from perfectly Mendelian. So we can now identify genes — as stretches of DNA — in cases where the original Mendelian means of identification do not apply. Knowledge of the natural property underlying the phenomenon picked out by our initial means of identification has allowed us to generate new means of identification which trump the original ones.

That is just the familiar story about how we can move beyond our initial means of identifying a natural property. Phenomenal properties may be disanalogous in various ways. But it shows that there is no good general argument that our initial means of identifying a property must be the most secure on pain of undermining the inquiry.

### Probabilistic Treatment

Another way to see the beguiling thought is to express it in terms of probabilistic degrees of belief. We must start the inquiry with a high degree of belief in A_p_ as evidence of P_p_. The empirical study is founded on identifying instances P_p_ and ¬P_p_ via behavioural evidence that is caused by A_p_ (we are supposing). So we must start with Pr(P_p_|A_p_) and Pr(¬P_p_|¬A_p_) being high. The inquiry then proceeds to identify some further property T_p_ that is common to most of the so-judged P_p_ instances and distinctive of them by comparison to the non-P_p_ instances (the test T_p_ may be a complex conjunctive property). So we come to have Pr(P_p_|T_p_) and Pr(¬P_p_|¬T_p_) both high. The beguiling thought is that we should not reach that conclusion at the cost of abandoning our belief in the probative value of A_p_. So we must continue to think that Pr(P_p_|A_p_) and Pr(¬P_p_|¬A_p_) are both high (correlatively, that Pr(¬P_p_|A_p_) and Pr(P_p_|¬A_p_) are both low). Giving those up would be to undermine the basis on which we predicated our study, leaving no reason for our confidence in test T_p_ to be high (i.e. for Pr(P_p_|T_p_) and Pr(¬P_p_|¬T_p_) to be high).

That is all very well. But what about the crucial issue: is it coherent to think that T_p_ trumps A_p_? Here the focus is on the degree of belief in P_p_|(T_p_&¬A_p_) and in ¬P_p_|(¬T_p_&A_p_). The beguiling thought is that these must be *low* since our degree of belief in P_p_|¬A_p_ and in ¬P_p_|A_p_ must be low. So the question, from a probabilistic perspective, is whether the following can form a coherent set:

**Table:** 

Initial evidence:	Pr(P_p_|A_p_) high	Pr(¬P_p_|¬A_p_) high
equivalently:	(1a) Pr(¬P_p_|A_p_) low	(1b) Pr(P_p_|¬A_p_) low
Further test:	(2a) Pr(¬P_p_|¬T_p_) high	(2b) Pr(P_p_|T_p_) high
Trumping:	(3a) Pr(¬P_p_|{¬T_p_&A_p_}) high	(3b) Pr(P_p_|{T_p_&¬A_p_}) high

The beguiling thought is that (3a) conflicts with (1a) and (3b) with (1b). Tempting though that thought is, it is wrong. It can be perfectly rational to distribute subjective probabilities in accordance with (1a), (1b), (2a), (2b), (3a) and (3b). The distribution of P_p_ in the A_p_ and ¬A_p_ regions required by (1a) and (1b) does not by itself constrain the distribution of P_p_ in the {¬T_p_&A_p_} and {T_p_&¬A_p_} regions. You can think that A_p_ is a very good sign of P_p_ except in the presence of ¬T_p_. So the empirical enquiry can conclude that in some cases T_p_ trumps A_p_ as a sign of P_p_, while holding on to A_p_ as a good sign of P_p_. The Appendix offers a proof.

A further consideration is also relevant here. You might think that, although Pr(P_p_|A_p_) and Pr(P_p_|T_p_) could both be high, Pr(P_p_|A_p_) must always be higher than Pr(P_p_|T_p_), since Pr(P_p_|A_p_) being high formed the basis on which we came to think that T_p_ is evidence for P_p_. But that is a bad thought too. We might only have moderate confidence in Pr(P_p_|A_p_) and still use A_p_ to generate a large sample of putative instances of P_p_. Then we may have a very high confidence that *most* of them are instances of P_p_; in particular, our confidence in that may far exceed our Pr(P_p_(S)|A_p_(S)) for a single case. If we then use that large sample to generate the new test T_p_, it may well be that T_p_ turns out to be a better test of P_p_ for a single case, so that Pr(P_p_(S)|T_p_(S)) > Pr(P_p_(S)|A_p_(S)).

That shows the consistency of a synchronic set of degrees of belief about the distribution of P_p_, A_p_ and T_P_. However, the real question, of course, is how the degrees of belief we have in P_p_|A_p_ and ¬P_p_|¬A_p_ at the outset of the inquiry (both high) constrain the posterior degrees of belief we can assign to ¬P_p_|(¬T_p_&A_p_) and P_p_|(T_p_&¬A_p_). Since we have shown the coherence of a *synchronic* set of degrees of belief according to which these are all high, there can be no belief-revision story according to which having high priors for P_p_|A_p_ and ¬P_p_|¬A_p_ prevents us arriving at high posterior probabilities for ¬P_p_|(¬T_p_&A_p_) and P_p_|(T_p_&¬A_p_).

To summarise, although the beguiling thought sounds plausible, when we are studying a natural property there is no general reason why our initial means of identification should restrict the inquiry in that way. That is not a conclusive answer to the methodological challenge, but it defeats one version of the challenge and brings out what is at stake. If there is a problem about getting evidence that P_p_ is distinct from A_p_, it depends upon specific features of phenomenal and access consciousness. The supposed methodological problem must arise from some restriction on reasonable priors connecting A_p_, P_p_, our initial lines of evidence, and other potential tests.

We dismissed one such restriction in section 2— a formulation of A-consciousness according to which it is epistemically impossible to know about cases of P without A or the converse. But even if we are leaving it open that P_p_ and A_p_ are natural properties, there may be other reasons why consciousness is a special case. The next section identifies the most plausible candidate and the remainder of the paper shows how it can be overcome.

## 4. The Methodological Challenge

Standardly, when we investigate a natural property our initial means of identification are defeasible because they work by picking out properties that are not constitutive of the kind. We pick out water by its liquidity and transparency, but being liquid and transparent are not necessary properties of H_2_O (either metaphysically or nomologically). Block argues that there is a special problem if, at the outset of the investigation, we are uncertain whether our means of identification are like that:

‘The problem does not arise in the study of, for example, water. On the basis of the study of the nature of accessible water, we can know the properties of water in environments outside our light cone — that is, in environments that are too far away in space and time for signals travelling at the speed of light to reach us. We have no problem in extrapolating from the observed to the unobserved, and even unobservable in the case of water, because we are antecedently certain that our cognitive access to water molecules is not part of the constitutive scientific nature of water itself. … Few scientifically minded people in the twenty-first century would suppose that water molecules are partly constituted by our cognitive access to them (Boghossian 2006), but few would be sure whether phenomenal consciousness is or is not partly constituted by cognitive access to it. It is this asymmetry that is at the root of the methodological puzzle of phenomenal consciousness. [The issue is] whether the machinery of cognitive accessibility is a constitutive part of the nature of phenomenal consciousness.’ (Block 2007, p. 483)

The problem, supposedly peculiar to phenomenality, is that we are antecedently uncertain whether, if there is an information processing property A_p_, that property is partly constitutive of P_p_. Block’s challenge is to show that we can answer the empirical question of whether P_p_ and A_p_ come apart in any actual cases in the face of this antecedent uncertainty.

The passage quoted suggests a general phenomenon: antecedent uncertainty about a constitutive connection would always produce this kind of methodological difficulty. However, there are good examples of that kind of uncertainty being overcome in other cases. For example, the atomic number of Californium was amongst the initial means by which instances of that element were identified, but that didn’t stop much more being learnt about the element once it had been synthesised (its atomic structure, the half-life of its various isotopes, etc.). So it doesn’t seem to be a problem if one of the initial means of identification is constitutively connected to the property being investigated. Indeed, if we were to start again in our study of gold but to add its atomic number to the familiar list of identifiable properties, then it is not obvious that problems would ensue. Identification by atomic number would be up for revision in the light of subsequent evidence, just like all the other means of identification, but wouldn’t end up being revised. These are cases where we could be antecedently uncertain but then come to discover that one particular property used as evidence was always co-instantiated with the property under investigation.

There are also cases where antecedent uncertainty has been resolved against the means of identification being constitutive. If you want to know whether two individuals are members of the same species, similarities and differences in their DNA are a very good guide. Indeed, having a genome with certain DNA properties could have been constitutive of species membership. For some time that was a live theoretical option. It turned out to be false. There is still debate about the best way of understanding species, but on no view are genetic properties now thought to be constitutive of species membership. The best account of species membership is that it is a matter of standing in an historical relation of common descent to your conspecifics — belonging to a given clade. So there was antecedent uncertainty about whether genetic properties were constitutive of species membership, as well as being a means of identifying species membership. Subsequent investigation revealed that they are not.

There may be important disanalogies between these cases and phenomenal consciousness. What they show is that the problem Block identifies, which doubtless does present methodological difficulties, can be overcome in other areas of science. We argue below that the approaches used in other areas can also work with phenomenal consciousness.

A preliminary disanalogy is the mind-dependence of the psychological states involved. With Californium and biological species we are working with mind-independent means of identification of an uncontroversially mind-independent natural property. Is it right to think of the “means of identification” we use for picking out instances of P_p_ in the same way? We argue that it is.

Both P_p_ and A_p_ are mind-dependent properties in the sense that they are mental properties. Are they mind-dependent in the sense at issue in literature on secondary qualities and response dependence? There are two candidates for the response on which the property being identified putatively depends: the initial lines of evidence and property A_p_ itself. Taking the first, it could turn out that there is nothing more to property P_p_ than a subject’s disposition to make a verbal report that she sees/hears/… that p. Taking the second, it could turn out that there is nothing more to instantiating P_p_ than the subject’s having the information that p globally available (i.e. being in state A_p_). The question of response dependence asks whether either of these is constitutive or partly constitutive of P_p_. That is simply to ask whether one of the means of identification is constitutively connected to P_p_.

In section 2 we set aside a priori response dependence (Wright 1988). If we are leaving it open as an empirical possibility that P_p_ is a verification-transcendent natural property, then we should treat the initial lines of evidence and A_p_ as some properties by which we identify others. A subject’s verbal report is a means by which we identify that she is likely to instantiate P_p_. Similarly gathering other evidence (beyond the initial lines of evidence) that a subject has property A_p_ (e.g. recording a pattern of coherent neural firing indicative of the global availability of information) is a means of identifying that she is likely to instantiate P_p_.

In the secondary qualities debate, we ask whether being red is a property possessed by the surface of objects independently of their relation to human experiences, or whether being red is a surface’s disposition to produce a certain kind of visual experience in human observers (in certain circumstances). Confusingly, the observer’s experience of red in the secondary qualities debate corresponds to A_p_ in our debate. P_p_ corresponds to the putative property of the surface.^5^ The question is whether there is such a property which is independent of A_p_. Just as the failure to find a surface reflectance property to identify with being red motivates a secondary quality account of colour, the failure to find a property independent of A_p_ to identify with P_p_ motivates an account of phenomenality according to which A_p_ and P_p_ are constitutively connected (and thus always co-instantiated). Asking a response-dependence-type question about P_p_ is just to ask whether A_p_ (or any of the other means of identification) is constitutively connected to P_p_. Whether it is or not, P_p_ is clearly a psychological property. But if P_p_ is studied as a natural property, P_p_’s being psychological poses no special issue as far as response dependence is concerned.

It is A_p_’s being a psychological property that gives rise to the response dependence question, but that is simply the question of whether one of the means of identification of P_p_ is constitutively-connected to P_p_. In this section we argued that there is no general problem with overcoming antecedent uncertainty about that. The task of the next section is to show that the way that uncertainty is surmounted in other areas of science is equally applicable to the study of phenomenal consciousness.

## 5. Application of the Natural Kind Methodology

We argue that the methodological problem is relatively tractable provided P_p_ is a natural kind: a natural property that supports a wide range of inductions. Our suggestion is that science should investigate the natural kinds that are responsible for generating and unifying the various behaviours that we take to be evidence of P_p_. The rough idea is that, if we find only one underlying natural kind, that is evidence that A_p_ and P_p_ are always co-instantiated. (In the same way as in the secondary qualities debate the absence of a second property with which to identify redness leads to the view that redness is constitutively connected to our means of identifying redness.) If we discover two underlying natural kinds, that is some evidence that P_p_ and A_p_ come apart, although that case is more complicated and is developed further in section 6.

Our account is in the same spirit as Block (2007), in that it relies on inference to the best explanation. However, it says more than Block does about the structure that inference will take and how to generate the appropriate data. Block’s inference is mainly based on his interpretation of an experiment by Landmann et al. (2003), a refinement of Sperling (1960) that uses an array of oriented rectangles. He argues that those experiments show that the capacity of phenomenality exceeds the capacity of global availability (different capacity → non-identity). Block also points to a ‘mesh’ between psychological and neuroscientific data, but the psychological data bear most of the weight. We argue that a more satisfactory answer to the empirical question will require a larger variety of sources of evidence. The suggestion is not just that there should be more data. The crucial point is that the data should be generated and interrogated in a particular way.

### Natural Kinds in Psychology

Discovering a natural kind allows us to move beyond our initial means of identification. We will see how that process works in science in general, and in particular in psychology, before going on to apply it to the particular case of consciousness.

Psychology has discovered many natural kinds, with a variety of substrates, but two examples will have to suffice. The first is the collection of language deficits that can be produced by brain damage. A great variety of deficits was observed before it was discovered that patients fall into various groups. One group has good language comprehension but non-fluent agrammatical speech. Another group has poor language comprehension but produces fluent seemingly grammatical speech. It was discovered that the first group forms a natural kind unified by the existence of damage to areas of the left ventrolateral prefrontal cortex (Broca’s area) and underlying areas. The second set of behavioural symptoms cluster together in virtue of a common mechanism of damage in the left posterior temporal cortex (Wernicke’s area). (There are further groups, e.g. conduction aphasia, and refinements of these groups.) In both cases there is an underlying natural property that gives rise to the set of behavioural symptoms and underpins inductions from case to case.

Sleep research furnishes an example in normal subjects. Evidence is gathered about properties of sleepers (heart rate, respiratory rate, eye movements) and their behaviour (how they respond to stimuli, what they say when they wake up). Such symptoms form a number of clusters, each associated with a different underlying state of brain activity as measured by EEG. The clusters support inductions from case to case (e.g. a slow-wave EEG trace predicts a lower heart rate and various other behaviours). Although the mechanisms have not yet been characterised fully, there is good evidence that REM sleep, say, forms a natural kind that is unified by an underlying causal process in the brain and body.

The natural kind methodology, as we will call it, is to gather together as many putative tests of a phenomenon as possible, ranging widely across types of evidence collected. It then interrogates the data to look for “nomological clusters”:

Nomological cluster

A set of evidential properties T_i_ form a nomological cluster iff

(i) they are instantiated together better than chance (given background theory); and
(ii) observing subsets of the cluster supports induction to other elements of the cluster.

A nomological cluster supports inductive inference from some of the T_i_ to others: e.g. a patient identified as a Wernicke’s aphasic behaviourally is likely to have damage to the left posterior temporal cortex. It also supports inductive inference to new cases: e.g. if we discover that a group of Broca’s aphasics show little semantic priming, we expect that a new patient with good comprehension and non-fluent agrammatical speech is likely to show little semantic priming. If the success of those inductive inferences is not pure chance it is because there is some natural reason why the properties over which we induce tend to cluster together. If so we can say they form a natural kind. The property in virtue of which they so cluster can also be called a natural kind or a natural kind property. A nomological cluster can be explained by the existence of a natural kind to which the T_i_ are nomologically related causally or constitutively.

Philosophers mean several different things by ‘natural kind’. A Lockean approach restricts the term to cases where some inner intrinsic essence is responsible for the identifiable symptoms (Putnam 1975). That would exclude cases where the property explaining the cluster was extrinsic. For example the property of being a member of a given biological species is partly an historical property, with similarities in surface properties explained by the fact that conspecific individuals are related by a process of descent, involving conservative copying of features, from the very same individual (i.e., by being members of the same clade). We are adopting a much broader conception of natural kinds according to which any natural property that supports induction as a result of nomological principles or natural laws counts as a natural kind (Hacking 1991, Griffiths 1997, Millikan 2000). That would include the cladistic property of being a member of a given biological species. For a natural property to be a natural kind is then a matter of degree, depending upon how broad and various are the properties over which it supports inductions. For the purpose of answering the methodological challenge, it doesn’t matter whether P_p_ is intrinsic or extrinsic, just that it is a natural property that supports a range of inductions.

Provided P_p_ is a natural kind within that broad family it will underpin the success of the inductive and explanatory practices in which our concept of P_p_ is deployed, even if the only means of identifying instances of P_p_ are imperfect. Granted, if false positives and/or false negatives are widespread enough, those inductive practices will fail. But they can succeed well enough to be useful in the face of some misidentifications.

With some of the concepts used in science it is part of the conception associated with the concept that the referent is whatever property plays a certain explanatory role. However, in order to pick out a natural kind, it is not necessary that a concept be explicitly a theoretical or explanatory role concept. We perform inductions over many properties which we simply identify and co-project, without it being part of the structure of the concept that the referent is whatever property plays a certain explanatory role. In our view, reference to the natural kind in these kinds of cases is established just in virtue of the fact that a natural kind in fact underpins the success of the inductive practices of concept-users deploying those concepts (Millikan 2000).

For example, even if water is not an explanatory role concept, it remains true that the reason we can project observed properties of water (transparency, action as a solute) to new cases is because of the electron shells of hydrogen and oxygen atoms and of how they combine (i.e. their chemical properties). The property of dissolving sugar, observed true of one body of stuff identified as odourless, colourless, transparent, potable liquid, carries over and is true of other bodies of stuff identified as odourless, colourless, transparent potable liquids because they are H_2_O. That these considerations are sufficient for reference to a natural kind is a substantive assumption, but it will be true on quite a variety of semantic theories, and it is something that ought to be a constraint on, or at least a desideratum for, any theory of the reference of these concepts. (Shea 2007, pp. 254-5 argues for this sort of constraint in giving a theory of the content of more low-level representations, below the level of concepts.)

### Studying P_p_ as a Natural Kind

The first stage is to investigate cases that we take, relying on current evidence, to be instances of P_p_, and to find further properties that are common to and distinctive of most of those instances. Some of this data may concern particular neural processes, like the presence of synchronic neural firing in the 40 Hz waveband or locally recurrent activation. It may also concern particular neural structures, like cortico-thalamic loops or cortical networks that integrate prefrontal cortex with other cortical areas. Some may be characterised in terms of information processing properties, others not. (The methodological sceptic, of course, will claim that these are all just properties of the A_p_ mechanism; or, at best, of the mechanisms of A_p_ and P_p_ combined.)

Another source of evidence will concern what phenomenal consciousness does for us — it will look for ways of characterising the functional profile of the instances taken to fall within our conception of P_p_. One example makes use of two different ways of conditioning the eye blink response. It seems that ‘trace conditioning’ requires phenomenal consciousness whereas ‘delay conditioning’ does not. In delay conditioning a puff of air to the eye is administered during the occurrence of a tone (after the start of the tone, hence ‘delay’ conditioning). Delay conditioning dissociates from subjects’ reports about the contingency between tone and air puff (Perruchet 1985). In trace conditioning the air puff occurs shortly after the tone has stopped. Unlike delay conditioning, trace conditioning seems to depend upon the subject’s being able to report the contingency between tone and air puff (Clark et al. 2001, Clark and Squire 1998, Perruchet et al. 2006). So trace conditioning correlates with and delay conditioning dissociates from phenomenal consciousness (as measured by verbal report).

The functional signature of P_p_ can be probed in a large variety of ingenious ways (Jack and Shallice 2001 defend a similar method and give examples). For example, ‘negative stem completion’ seems to go with phenomenal consciousness (Debner and Jacoby 1994). Subjects are briefly shown a written word, followed by the first three letters of that word. The task is to complete the stem to make any word other than the word originally shown. For example, shown frigid then fri the subject could say ‘fright’; ‘frigid’ would be an incorrect response. Masking is used to manipulate whether subjects are phenomenally conscious of the original word. When subjects do not consciously see the original word, they tend to respond incorrectly, that is they use the original word to complete the stem (an unconscious priming effect). Only when phenomenally conscious of the original word do subjects avoid that tendency (see Merikle et al. 2001 for a review). Another task that appears to require phenomenality is making discriminations among novel conjunctions of perceptual features. To do this, the subject may need first consciously to experience a stimulus displaying that conjunction (Jack and Shallice 2001, p. 174).

Candidate functional tests are ones which either seem to require phenomenal consciousness, or which are performed in a different way when the relevant parameters form part of the subject’s phenomenal consciousness,^6^ so that the mechanism deployed when performing the task relying on phenomenality has a different functional signature from the non-phenomenal mechanism for performing the task. There are many other candidates: recombining and re-using elements of a plan, spontaneous generation of behaviour, spontaneously noticing a feature of an unchanging perceptual stimulus, etc.

Some tests seem closely connected to global availability (e.g. investigating consciousness in animals via meta-memory: Hampton 2001 and Shea & Heyes 2010). Others like the trace conditioning example above are not obviously connected to global availability (although they may turn out to depend upon it, if we discover that P_p_ and A_p_ are always co-instantiated). There is no obvious reason why trace conditioning should, but delay conditioning should not, depend upon global availability of information. So far we know just that trace conditioning comes with phenomenality. That may be because it comes and goes with A_p_, but if P_p_ and A_p_ do in fact dissociate in actual cases, it could equally well be that trace conditioning comes and goes because of the nature of P_p_, irrespective of its connection to A_p_.

The following list gives some examples of the sorts of tests that can be deployed in investigating P_p_ as a natural kind. Only those in group (a) seem to be directly dependent on A_p_:-

Putative tests— T_i_

(a) Connected to global availability of information:
  ○ Report of one amongst many features, cued after the stimulus has been masked, of a complex visual stimulus, Sperling (1960), Landman et al. (2003)
  ○ Meta-memory, Hampton (2001)
  ○ Insensitivity to the automatic stem completion effect, Debner & Jacoby (1994), Merikle et al. (2001)
  ○ Integration of information by prefrontal cortex
(b) Connected to information processing but not necessarily global availability:
  - Susceptibility of grip width to the size contrast effect, Hu & Goodale (2000)
  - Trace conditioning (vs. delay conditioning), Clark et al. (2001)
  - Perceptual discrimination of a novel conjunction of perceptual features, Jack & Shallice (2001)
(c) Not characterised in information processing terms:
  ▪ 40 Hz / gamma-band neural synchrony
  ▪ Local recurrence in cortex
  ▪ Cortico-thalamic loops
  ▪ Networks involving prefrontal cortex

Once there is a wide variety of data we can look for nomological clusters. For our purposes the important question is whether there is one cluster or two (or more). To assess that requires some sophisticated causal modelling of the network of connections between the various tests T_i_ (Pearl 2000). That is by no means straightforward. Nor is it merely a mechanical process. Application of causal models relies on various assumptions, some of which are contentious when considering the metaphysics of causation. However, legitimate questions about the suitability of these models as accounts of causation do not undermine the reliance we place on them here, which requires only that there is a viable epistemology of causation: a way of gathering evidence as to what the causal relationships are amongst various properties that have been empirically observed across a variety of instances and of inferring the existence of underlying causes.

### One Cluster

**Figure 2:**
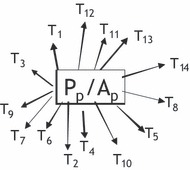
P_p_ and A_p_ always co-instantiated

If we find only one cluster amongst the T_i_, then we will have discovered good evidence that P_p_ and A_p_ are always co-instantiated: [Fig. 2]

The arrows in the diagram represent causal effects of A_p_. For example, it could turn out that the global availability of information is achieved by gamma-band neural synchrony across long-range cortical networks that allows information in the back of the brain to be integrated in the prefrontal cortex. That would be our natural kind A_p_. Our causal modelling might show that A_p_ itself was the most likely cause of verbal reports etc. (our initial tests), and of the symptoms subsequently found to covary with those tests (e.g. trace conditioning, gamma-band EEG traces, etc.). If no other property were discovered underlying the co-projection of all the tests T_i_, then that would be good evidence that P_p_ is identical to A_p_, or co-instantiated as a matter of at least nomological necessity.

A sceptic might claim that it is still possible that P_p_ and A_p_ are only causally connected, but that A_p_ causally screens off our access to P_p_, so that the only way we can detect the presence of P_p_ is via detecting A_p_. (That would be a causal analogue of the beguiling thought — that A_p_ screens off our epistemic access to P_p_— dismissed in section 3.) However, if P_p_ and A_p_ are different natural kinds, with P_p_ causing A_p_, it is implausible that P_p_ should be causally inaccessible except via A_p_. The initial tests may depend causally on A_p_ and thereby ensure that P_p_ is always co-present with A_p_ in our initial sample, but if there are two natural kinds, then both properties are present in our in-group and both properties are absent in our out-group. If we investigate all the effects of the properties in our in-group we will get downstream consequences of both P_p_ and A_p_. Effects of both will be absent from the out-group. As a separate natural property P_p_ will have causal consequences that do not proceed via A_p_. The methodological challenge is that it is difficult to tell which are which, not that P_p_ is totally causally isolated behind A_p_.

By analogy, consider the comedy duo Pen and Teller. Pen is always silent. If you shut your eyes, your only evidence about whether Pen is present is the testimony of Teller. Nevertheless, Pen is not causally screened off, so you can rely on Teller to say when Pen is there and then use other tests to confirm Pen’s independent existence (e.g. touch or sight). If Pen is a separate person it is implausible that all his actual and potential causal effects on the world should proceed via Teller.

On any view, brains will play an important role in instantiating P_p_. And if P_p_ causes A_p_ it is overwhelming likely that P_p_ is instantiated before A_p_ in time. But there is no reason to suppose that the goings on in human brains before A_p_ is instantiated will have no causal consequences except to give rise to state A_p_. All the usual brain measures have the potential to detect P_p_ directly (EEG, MEG, PET, fMRI, etc.), and there will likely be functional tests too. So the causal isolation idea is a non-starter.

### Two Clusters

So much for the conclusions we would reach if the empirical investigations uncover only one underlying natural kind. Our method may instead discover that there are two natural kinds underlying instances to which our concept of P_p_ was initially applied, one of which is actually instantiated separately from A_p_ on some occasions.

For instance, suppose we discover a mechanism of global availability as before: gamma-band neural synchrony across long-range cortical networks connecting the prefrontal cortex with posterior areas of the brain (Kind2). Suppose we also discover a second underlying kind that is also common to most of the instances in our initial sample. It might be a more local kind of connectivity: local cortical-cortical resonance sustained by cortico-thalamic loops (Kind1). (To contrast with the A_p_ mechanism we can suppose that the cortico-thalamic loops are ‘vertical’ and so do not sustain strong interactions between distantly-separated cortical areas.) What do we say about someone who has colour information resonating in a local cortical-cortical circuit sustained by a vertical cortico-thalamic loop (Kind1) without that information being made globally available via long-range neural synchrony (Kind2 / A_p_)? Since Kind1 and Kind2 were co-present in our initial sample (verbal report etc.), either could have been the property in virtue of which those subjects were conscious. The fact that A_p_ is absent in this particular case should not lead us to conclude that P_p_ is absent (since we have ruled out tying P_p_ to A_p_ a priori), and the fact that another natural kind underlying the original samples is present (Kind1) gives us some evidence that this kind is P_p_.

**Figure 3:**
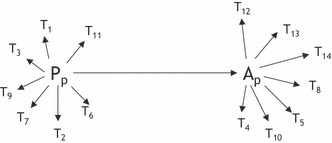
P_p_ causing A_p_ generates two clusters of properties T_i_.

To illustrate, we may find that Kind2 / A_p_ is the cause of verbal report, meta-memory and gamma-band EEG traces, and that Kind1 is the cause of trace conditioning, susceptibility of grip width to the size contrast effect, and the ability to detect novel conjunctions of visual features. In normal cases Kind1 causes Kind2, so these tests are normally all present or all absent (provided co-operating mechanisms like language are not otherwise disabled). But in unusual cases they come apart, which allows us to see that there are two clusters of tests, each underpinned by a different underlying kind. Figure 3 illustrates the usual case where P_p_ causes A_p_. In the rarer cases where only P_p_ is present only the T_i_ on the left would be found. The T_i_ on the right would be absent.

If P_p_ and A_p_ are in fact two kinds which come apart in actual cases, then casting our net of tests widely is likely to encompass some instances where P_p_ occurs in the absence of A_p_, and so deliver evidence that P_p_ is a separate natural kind. Correlatively, if a wide empirical enquiry of the sort suggested here furnishes a large number and variety of tests T_i_ that cluster on a single underlying natural kind, that is good abductive evidence that A_p_ and P_p_ are always co-instantiated.

**Figure 4:**
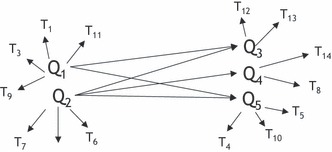
Causal modelling of the T_i_ could uncover many clusters with complex interrelations.

Of course, the clustering of the T_i_ may show that neither empirical option we were considering is correct. We leave for another day the question of what to say about phenomenality if it turns out that there are many different natural kinds (or none). For present purposes, it is enough to observe that, as well as deciding between them, the natural kind methodology is capable of delivering evidence that neither of the hypotheses between which we are trying to decide is true Fig. 4.

For completeness we should notice that the methodology will give rise to trumping. Whether we find one cluster or two clusters, the T_i_ will be stronger than the original evidential tests (verbal report and the like), perhaps individually and certainly collectively. So if it turns out that there are two underlying natural kinds, the tests T_i_ will allow us to pick out cases of P_p_ without A_p_, even if the original means of identification of P_p_ would have told us that they were not cases of P_p_.

## 6. Further Lines of Evidence

### 6.1 The pre-P Question

If the evidence shows that there is only one underlying natural kind, then we have good reason to conclude that P_p_ and A_p_ are at least nomologically co-instantiated. That is the easier case. A discovery of two underlying kinds would raise a further question.

**Figure 5:**
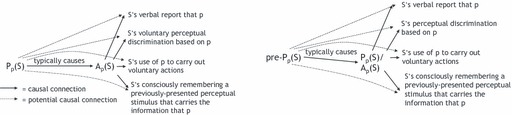
Competing hypotheses given two underlying kinds.

If we discover two natural kinds how do we know that the first kind really is phenomenal? There is an alternative picture according to which P_p_ and A_p_ are still always co-instantiated and Kind1 is a pre-phenomenal natural kind that is a normal causal antecedent to the P_p_/A_p_ kind. That alternative has not been conclusively eliminated, but there is further evidence that can be brought to bear. The task is to gather evidence to choose between the following two competing hypotheses illustrated in Fig. 5 below.

The first thing to say is that, even if the hypothesis of pre-P_p_ causing P_p_/A_p_ has not been ruled out, the likelihood of the hypothesis that P_p_ causes A_p_ has increased, since one of the ways that it could have been false, namely if there had been only one underlying natural kind, has been ruled out. But although its likelihood would have gone down, discovering two underlying natural kinds would not rule out the possibility that Kind1 is pre-P_p_ and not P_p_. We address the issue in two parts, distinguishing between first-personal and third-personal ways of identifying P_p_.

First-personally, we can each know what we are phenomenally conscious of in a very direct way in virtue of being phenomenally conscious in that way (that knowledge need not be infallible or incorrigible). I am somehow able reliably to apply a phenomenal concept of P_p_ to myself directly when I instantiate P_p_, without making use of any of the behavioural signs of consciousness. Third-personally, we identify instances of P_p_ in others through identifying various other properties (T_i_). (We can also identify P_p_ in ourselves in these third-personal ways.) We start by taking our concept of P_p_ to have only third-personal modes of application. Section 6.3 discusses the purely first-personal concept and 6.2 the mixed case.

To assess whether Kind1 is P_p_ we inevitably have to take a stand on issues about the semantics of the concept of P_p_. Detailed treatment of these issues is beyond the scope of the present paper, so we appeal here to the plausibility of the principle above about the reference of concepts that are relied on for co-projection and induction. If our concept of P_p_ has only third-personal modes of application, then we can rely directly on the argument above. We argued that irrespective of whether we conceive of P_p_ as being the occupant of a functional role, our concept refers to whatever property underpins the successful inductions in which it is deployed. By hypothesis, Kind1 underpins some of those inductions. For example, in the illustration from the last section Kind1 is local recurrence and the induction from trace conditioning to verbal report proceeds via Kind1, as does the induction in the opposite direction, from meta-memory to susceptibility of grip width to the size contrast effect. Our large battery of tests normally cluster together because Kind1 and Kind2 normally come and go together. Some of the clustering depends on direct causal connections of some of the T_i_ to Kind1. So P_p_ refers to the basis of that clustering, namely Kind1.

An objector might suggest that, even if some scientific tests depend on Kind1, all commonsense inductions about being phenomenally conscious proceed only via Kind2 / A_p_. But that is implausible if Kind1 and Kind2 are normally co-present. For example, we have a lot of everyday knowledge about causal interventions that will abolish the phenomenal property of having a pain in the foot. We can take codeine, undergo acupuncture, re-direct our attention or breathe deeply. Even if some of those routes work just by abolishing A_pain_ it is implausible that they all should. Once we accept the point above about P_p_’s not being causally screened off (the Pen and Teller example), if P_pain_ and A_pain_ are co-present when we make ordinary inductions about pain, say, it is very unlikely that A_pain_ should be responsible for the success of all those inductions, with none relying causally on the presence of P_pain_.

This strategy would be particularly persuasive if it were to turn out that one of the initial lines of evidence is sometimes directly caused by Kind1 without the mediation of A_p_. That possibility is represented by dotted arrows on the diagrams above. For example, it may turn out that Kind1 sometimes directly causes information to be stored in episodic memory, even when it is not globally available. It might even turn out that some voluntary verbal reports of the information that p are made without that information being globally available (although presumably it would become available shortly afterwards, if the subject listens to their own report). If S evinces one of the initial lines of evidence due to instantiating A_p_ then she should, barring some interfering factor, evince all the others too. However if the storing of a particular episodic memory, say, were due only to instantiating Kind1, without A_p_, then she would not show the other signs (verbal report, etc.) at the time, despite the other consuming systems being unimpaired. Some of the inferences between memory storage and the other lines of evidence would then depend causally on Kind1.

If that is how things turn out, then it would be reasonably clear that our concept of P_p_ would refer to Kind1, since even inferences amongst our initial standard means of identification depend on it. That is not the only way we could be convinced that Kind1 = P_p_— the general considerations about what underpins our inductive practices do not rely on it — but it would convince those whose semantic theory gives special weight to the initial means of identification.

### 6.2 Working with a partly first-personal, partly third-personal concept

The story is not substantially different if our concept of P_p_ is partly first-personal and partly third-personal. That is to say, as well as tracking phenomenality through our initial lines of evidence and other third-personal tests T_i_, we also identify instances of P_p_ in ourselves in an unmediated way, tokening of the concept being caused directly by instantiation of the property in the same subject.

Provided the first-personal modes of application have no special status, then they would just be further means of identifying instances of P_p_ (ones that the subject can only use on herself). Some inferences would depend upon third-personal ways of identifying P_p_, some on first-personal identification, and some would go from one to the other. Our argument that the concept refers to the property or properties that underpin those inductive practices still applies. If Kind1 and Kind2 come and go together, then it is very likely that Kind1 will causally underpin the success of some of those inferences. Even if all the first-personal identifications of P_p_ depended on A_p_/ Kind2, if first-personal uses are just further means of identification, on a par with the third-personal, the fact that some of the inductions rely on third-personal means of identification would be enough to ensure that Kind1 was needed to underpin the success of those inferences. So the concept of P_p_ would end up referring to Kind1.

In short, if the first-personal and third-personal ways of picking out P_p_ have equal standing, and application of the natural kind methodology uncovers two natural kinds underpinning our practices of making inductions over the cases so-identified, then we would have reason for thinking that Kind1 is the referent of our concept of P_p_.

### 6.3 Working with a Primarily First-Personal Concept

Things look different if the first-person mode of application is primary. Here we can imagine a sceptic who says, ‘I don’t care what natural kind is picked out by our ordinary third-personal ways of identifying phenomenality (verbal report and the like), what I want to know is whether instantiating property X would feel like *this*’— where the subject is herself instantiating the property of being phenomenally conscious that p. That is to take the first-personal mode of application as privileged — to think of P_p_ as picked out by what is sometimes called a phenomenal concept.

A first answer is that the ordinary concept of phenomenality does not work like that, nor should it. That answer is fully adequate, but it is instructive to see that, even if the sceptic is taken on his own terms, the natural kind methodology has more to offer.

The question is: what would be the reference of a purely first-personal phenomenal concept, which one applies to oneself directly in virtue of instantiating the experience P_p_, and only then? Would such a concept refer to Kind1, the natural kind we have discovered in addition to A_p_? Inductions made about other people must be set aside. Considerations about the inductions one makes in one’s own case over one’s own instantiation of P_p_ are now all that is relevant. It might be that, in respect of these inductions too, we would discern cases that rely on Kind1 as well as those that rely on A_p_. This subsection suggests the way further evidence about that can be gathered.

First we need to characterise first-person applications of the concept in more detail. What seems to happen when a subject acquires a phenomenal concept of the subjective character of one of her own experiences, is that she forms a disposition to classify together episodes of her experience in virtue of similarity in their phenomenal character (Loar 1990, Papineau 2002). If the episodes which she is disposed to classify together do indeed share some real property, then her phenomenal concept will succeed in referring — it will refer to that common property. The phenomenal property, though, is independent of her applications of the phenomenal concept. Indeed, it seems that subjects instantiate phenomenal properties of which they have never formed a phenomenal concept (quantifying over experiences in general — of course, if the subject starts thinking about any one such phenomenal property, she will somehow have formed a concept of it).^7^ Even when the subject does possess a phenomenal concept of a given phenomenal property she can have one of those experiences without applying the concept to it; and when she does apply the concept to it, application of the concept usually follows from (comes after) her instantiation of the phenomenal property to which it refers.

This bit of reflection makes plausible the claim that instantiation of the phenomenal property *being phenomenally conscious that p* is independent of, and typically causally antecedent to, first-person application of the concept being phenomenally conscious that p directly to oneself in virtue of having that experience. That is the extra bit of data that will help to address the semantic issue. Notice its modesty. The claim is not that we can introspect that there is phenomenality without access. Let us allow that phenomenal concept application is sufficient for A_p_: exercise of the concept being phenomenally conscious that p makes the information that p^8^ globally available for use in directing a range of behaviours (or requires that the information that p be so available).^9^

We make no such supposition about the property *being phenomenally conscious that p*. We are leaving open whether it is dependent on or independent of A_p_. As far as this line of argument goes, the property *being phenomenally conscious that p* may or may not always be co-instantiated with A_p_. If it is so connected, it remains separate from the property of *exercising the concept being phenomenally conscious that p*. Of course, we’ll need some lines of evidence that a subject is exercising the concept being phenomenally conscious that p. A lot is known about the cognitive psychology of concepts, and about the neural basis of the exercise of concepts. Gathering and applying such data presents no special methodological obstacles. So it is perfectly reasonable to suppose that we will be able to formulate good empirical tests for whether a subject is exercising the concept being phenomenally conscious that p, and that these will be independent of the battery of tests T_i_ by which we tell that a subject is *phenomenally conscious that p*.

To carry forward the enquiry from there, we would have to discover more about the nature of the property to which first-person uses of the concept are applied. Is its application directly triggered by Kind1, or is its application instead always mediated by A_p_? We will not speculate here about how that enquiry is likely to go. A more limited point is sufficient for our purposes, given our concern with potential methodological obstacles. If our introspective take on applications of the phenomenal concept is correct, then science should be able to distinguish between exercise of the concept and instantiation of the property to which it is applied. Those latter are the instances about which we need to ask. If they fall in the A_p_ cluster then we will have abductive evidence that P_p_ and A_p_ are nomologically co-instantiated, with Kind1 a pre-phenomenal property in the vicinity. On the other hand, if Kind1 is typically the direct causal antecedent to first-person exercise of the concept being phenomenally conscious that p, and people sometimes instantiate Kind1 without thereby instantiating property A_p_, then we will have abductive evidence that a purely first-personal phenomenal concept of being phenomenally conscious that p (if there were such a concept) would refer to Kind1, which is independent of A_p_.

This investigation can succeed even though, as we have conceded, phenomenal concept application is sufficient for A_p_ (things are only easier if not). However, the method does depend on phenomenal concept application not being necessary for A_p_. If it were, phenomenal concept application and A_p_ would always go together, making it impossible for empirical methods to tell them apart. However, the argument we made above about phenomenal concept application not being necessary for P_p_ also shows that it is implausible that phenomenal concept application is necessary for A_p_. A subject can have an experience of p and make the information that p globally available (i.e. instantiate P_p_ and A_p_) without having a phenomenal concept of that experience at all. The content of A_p_ may just concern the world, as may the content of P_p_ (if it has a content), whereas the content of the phenomenal concept concerns the thinker’s own psychological states. It is not plausible that having such meta-level concepts is necessary for object-level contents to be globally available (A_p_). Even where the subject happens to have a phenomenal concept of P_p_, tokening of that meta-level concept is not plausibly required for the object-level information that p to be made globally available.

## 7. Conclusion: a Methodology that will Encounter the Phenomenal Kind

If we think consciousness really could be a natural phenomenon, then we ought to be open to moving beyond our initial means of identifying instances of it. This paper has set out a systematic method for doing so.

Studying phenomenal consciousness as a natural kind presents no special obstacle unless it turns out that our ordinary ways of identifying P_p_ depend causally on a mechanism for the global availability of information A_p_. If we discover no such mechanism, then that is a very straightforward route to the existence of P without A.

Such an easy resolution seems unlikely because there is good evidence for the existence of an A_p_ mechanism (a global workspace). The method we then suggest is to perform a systematic search for the natural kind or kinds underlying the successful inductions that can be made about people who are phenomenally conscious. If we find only the A_p_ mechanism, and no second natural kind underlying those inductions, then that is strong evidence that P_p_ is always co-instantiated with A_p_. The systematic scientific study of consciousness has only just begun, but on the current and very partial state of the evidence that outcome looks to be worth a bet. What makes our approach significant is that it shows that the co-extensiveness of A and P can be something we discover empirically, rather than something that is built into the assumptions we bring to the enquiry.

Although it currently looks less likely, it could turn out that there are two natural kinds underlying the successful inductions involving P_p_, kinds which normally come along together because Kind1 causes Kind2, but which come apart in some actual cases. We have argued that this would give us evidence that P_p_ and A_p_ come apart in such cases since, on plausible assumptions, our concept of P_p_ would refer to Kind1 and A_p_ to Kind2.

One of the assumptions was that our concept of P_p_ is sometimes deployed using third-personal modes of application, and that any first-personal modes of application are in no way privileged. We think that assumption is correct, so the argument could end there. But we have gone on to show that, even if not, distinguishing phenomenal concept application from P_p_ and A_p_ would still give us a way of resolving the issue empirically.

If there do turn out to be two underlying natural kinds, then consciousness will fractionate in a way that goes against commonsense intuition. We could find subjects who are phenomenally conscious that p but without the information that p being *globally* available. The subject would show evidence of P_p_ in some but not all types of behaviour. For example, the information might be unavailable to verbal report. It seems odd that we could conclude that a person who reports not having an experience that p is nevertheless phenomenally conscious that p. But these would necessarily be odd cases, since the subject would evince the information that p in some modes of behaviour. The arguments in this paper show that, if we remain open to the possibility that phenomenal consciousness is a natural kind, scientific discoveries about it could give us good reason to revise our commonsense intuitions about such cases.

## Figures and Tables

**Figure 6:**
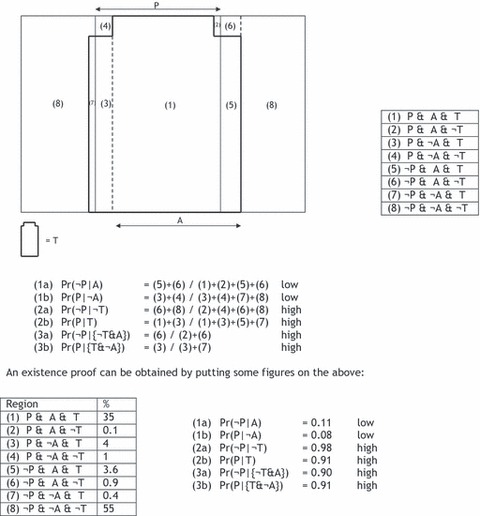
A coherent distribution of degrees of belief amongst P, A, and T.

## Footnotes

1 See the commentaries on Block (2007).

2 Experiments tend to rely on verbal reports with object-level contents (e.g. ‘There is now a red cube in front of me’) rather than so-called “subjective” reports with meta-level contents (e.g. ‘I see that there is a red cube’). In philosophical contexts, it is more common to rely on meta-level reports, because then the content of the verbal report is the same as the property we ascribe to S in reliance on her verbal report: that S has the phenomenal property, P_RC_, of being phenomenally conscious of a red cube; i.e. P_RC_(S). In ordinary talk, people move seamlessly between the two types of report. For our purposes, both kinds of report are evidence of the subject’s phenomenal state.

3 It is not straightforward to specify what it is for information to be ‘directly’ available. Roughly, the idea is that no further information-processing is required for it to be used by a consuming system (a formulation that is open to precisification in the light of unfolding data about the nature of the relevant psychological mechanisms).

4 Not all unencapsulated information gives rise to instantiation of a corresponding A_p_ property. If there are ‘central systems’ that solve the frame problem, then relevance-driven searches somehow cover our whole stock of beliefs without each of those beliefs thereby being available to a wide range of consuming systems.

5 See the right-hand diagram at the end of section 2 above.

6 For example, if consciousness significantly improves performance on a task (rather than being essential for it), or produces a distinctive pattern of errors, doing the task in the consciousness-involving way will thereby have a distinctive functional signature.

7 This does not rule out a species of representationalism according to which phenomenal experience has conceptual content; it just says that we can have such experiences without applying phenomenal concepts to them (phenomenal concepts are concepts *of* the experience).

8 Most obviously, it should make globally available the meta-level information that S is phenomenally conscious that p. But we can concede that global availability of the object-level information is a likely concomitant.

9 If not, we would be in even better shape methodologically, because we could then discern differences between three properties of S: A_p_(S), S’s exercise of the concept being phenomenally conscious that p, and S’s instantiation of the property of *being phenomenally conscious that p*.

## Appendix

The following distribution of degrees of belief is coherent:

**Table:**

Initial evidence:	Pr(P|A) high	Pr(¬P|¬A) high
equivalently:	(1a) Pr(¬P|A) low	(1b) Pr(P|¬A) low
Further test:	(2a) Pr(¬P|¬T) high	(2b) Pr(P|T) high
Trumping:	(3a) Pr(¬P|{¬T&A}) high	(3b) Pr(P|{T&¬A}) high

In particular, the distribution of P in the A and ¬A regions required by (1a) and (1b) does not, on its own, constrain the distribution of P in the {¬T&A} and {T&¬A} regions. That can be shown by the existence proof below, by assigning some values, but is perhaps most readily appreciated from a diagram: [Fig. 6]
